# The inflammatory spectrum of cardiomyopathies

**DOI:** 10.3389/fcvm.2024.1251780

**Published:** 2024-02-23

**Authors:** Nicolas Musigk, Phillip Suwalski, Ainoosh Golpour, DeLisa Fairweather, Karin Klingel, Pilar Martin, Andrea Frustaci, Leslie T. Cooper, Thomas F. Lüscher, Ulf Landmesser, Bettina Heidecker

**Affiliations:** ^1^ Deutsches Herzzentrum der Charité, Department of Cardiology, Angiology and Intensive Care Medicine, Berlin, Germany; ^2^ Department of Cardiovascular Medicine, Mayo Clinic, Jacksonville, FL, United States; ^3^ Department of Environmental Health Sciences and Engineering, Johns Hopkins Bloomberg School of Public Health, Baltimore, MD, United States; ^4^ Center for Clinical and Translational Science, Mayo Clinic, Rochester, MN, United States; ^5^ Cardiopathology Institute for Pathology, Eberhard Karls Universität Tübingen, Tübingen, Germany; ^6^ Centro Nacional de Investigaciones Cardiovasculares (CNIC), Centro de Investigación Biomédica en Red Cardiovascular (CIBER-CV, ISCIII), Madrid, Spain; ^7^ IRCCS San Raffaele, Rome, Italy; ^8^ GZO-Zurich Regional Health Centre, Wetzikon & Cardioimmunology, Centre for Molecular Cardiology, University of Zurich, Zurich, Switzerland; ^9^ Royal Brompton & Harefield Hospitals and National Heart and Lung Institute, Imperial College, London, United Kingdom

**Keywords:** myocarditis, inflammatory cardiomyopathy, heart failure, cardiomyopathy, endomyocardial biopsy (EMB)

## Abstract

Infiltration of the myocardium with various cell types, cytokines and chemokines plays a crucial role in the pathogenesis of cardiomyopathies including inflammatory cardiomyopathies and myocarditis. A more comprehensive understanding of the precise immune mechanisms involved in acute and chronic myocarditis is essential to develop novel therapeutic approaches. This review offers a comprehensive overview of the current knowledge of the immune landscape in cardiomyopathies based on etiology. It identifies gaps in our knowledge about cardiac inflammation and emphasizes the need for new translational approaches to improve our understanding thus enabling development of novel early detection methods and more effective treatments.

## Graphical Abstract

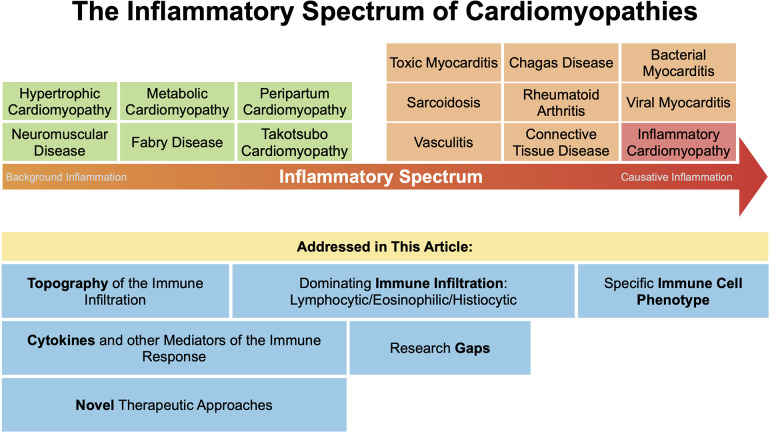

## 1 Introduction

**Figure 1:**
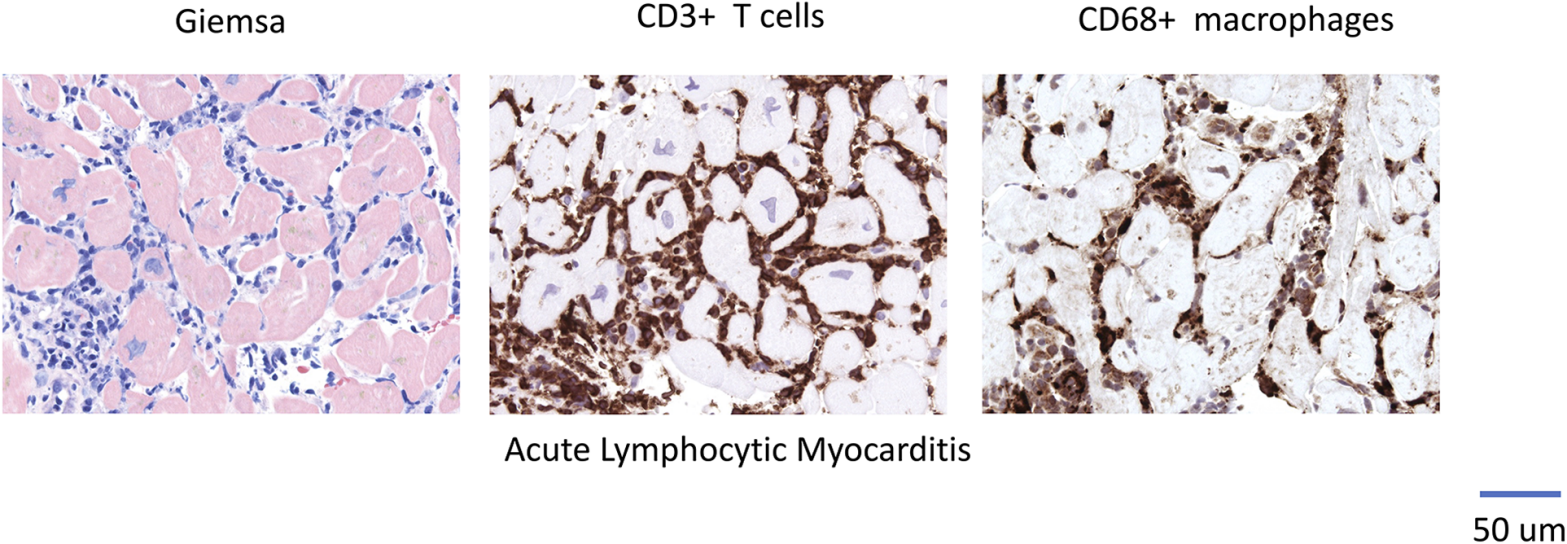
Representative EMB Findings in Acute Lymphocytic Myocarditis.

**Figure 2:**
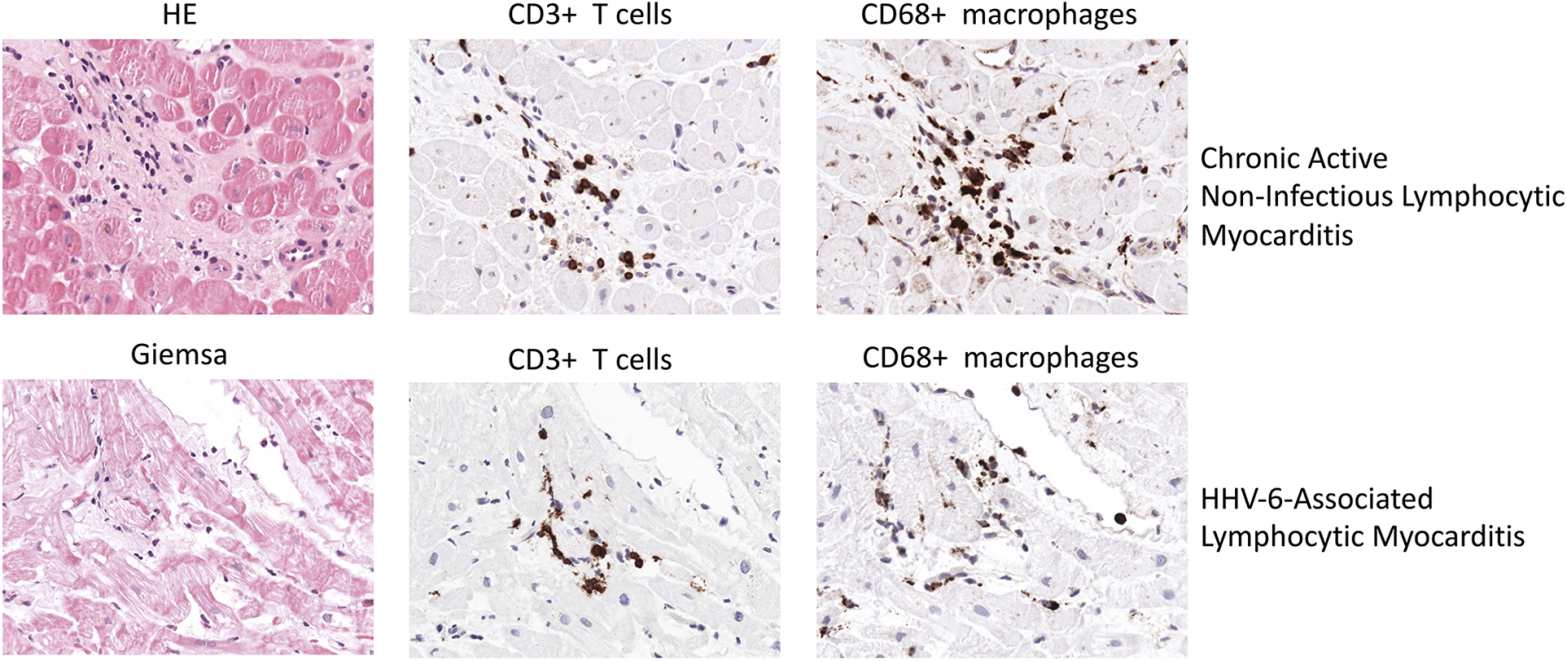
Representative EMB findings in chronic active Non-infectious lymphocytic myocarditis and HHV-6-assoziated lymphocytic myocarditis.

**Figure 3:**
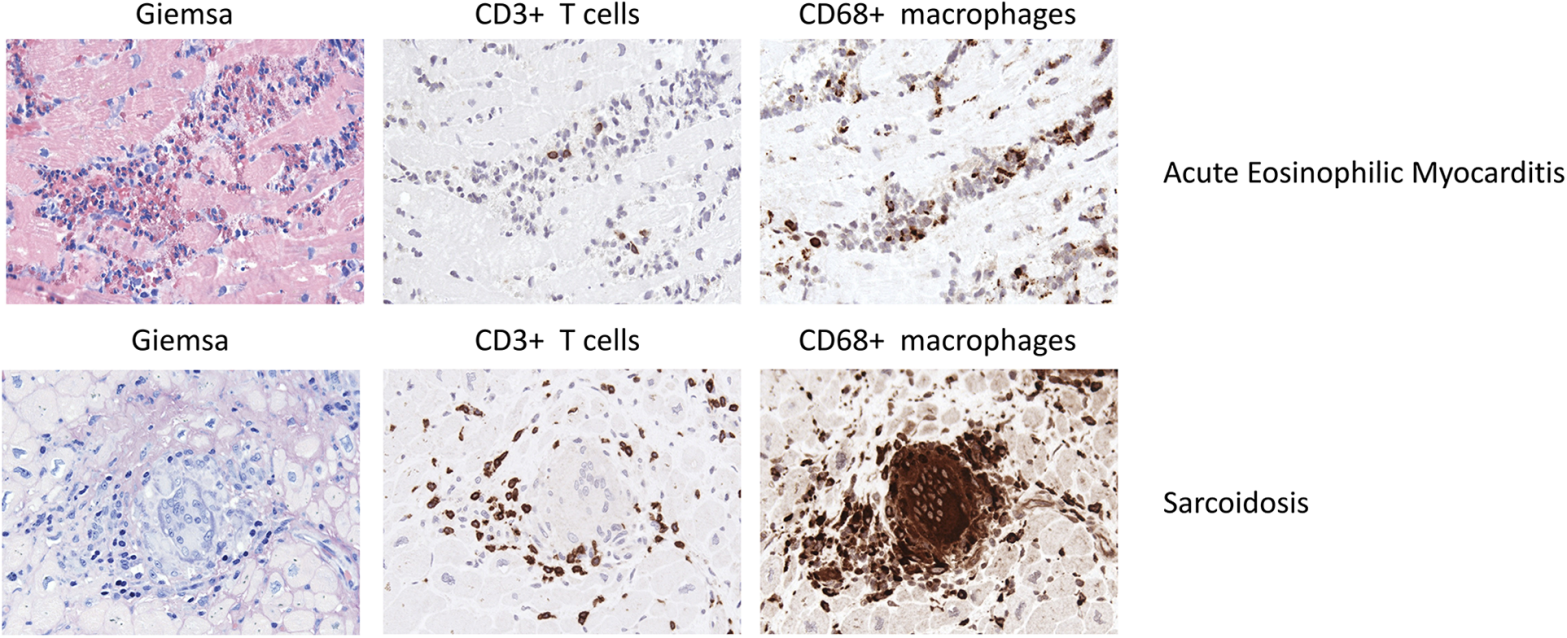
Representative EMB findings in acute eosinophilic myocarditis and sarcoidosis.

Cardiomyopathy may result from various etiologies associated with a reduction in left ventricular ejection fraction (LVEF) (1). Myocarditis is characterized by inflammation of the myocardium and can progress to chronic inflammatory cardiomyopathy or dilated cardiomyopathy (DCM) in susceptible individuals (2, 3). Immune cell infiltration (as illustrated in Figures 1–3 showcasing representative EMB findings), cytokines and chemokines play a central role in this process (2, 3). Resident mononuclear immune cells in the pericardium have been reported to amplify or regulate the heart-specific adaptive immune responses (4).

The precise prevalence of myocarditis remains uncertain. Myocarditis has been documented to occur in approximately 10–106 cases per 100,000 people globally (5). Patients suffering from cardiomyopathies may present with a spectrum of symptoms ranging from chest pain, dyspnea, palpitations or syncope to cardiogenic shock, in particular in the context of fulminant myocarditis (3, 6, 7).

Inflammatory cardiomyopathy may be triggered by many factors including infections, drugs, autoimmune conditions, and toxins and maintained through dysregulation of the immune system, which plays a critical role in the development and progression of the disease (8–11).

Viral infections are considered to be the most common cause of myocarditis, while numerous other etiologies of myocarditis and inflammatory cardiomyopathy have been reported (5, 12). Inflammatory infiltrates have been increasingly observed in endomyocardial biopsies (EMB) of various forms of non-ischemic cardiomyopathy including peripartum cardiomyopathy, takotsubo cardiomyopathy and cardiomyopathies associated with genetic variants of ion channels or structural proteins (8, 13, 14). Importantly, the release of proinflammatory and profibrotic cytokines, as well as other mediators by immune cells during acute myocarditis is critical for the progression from acute myocarditis to chronic inflammatory cardiomyopathy or DCM (15–21).

Recently there has been growing interest in the scientific community in immune cell-based and immunosuppressive therapies to restore immune homeostasis and to positively influence the clinical course at an early stage.

Gullestad et al. found that patients with congestive heart failure or idiopathic DCM that received intravenous immunoglobulin (IVIG) therapy produced elevated blood levels of the anti-inflammatory and anti-fibrotic mediators interleukin (IL)-10, IL-1 receptor antagonist and soluble tumor necrosis factor (TNF) receptor, leading to improved LVEF (22). It has also been shown in several studies that immunosuppressive therapy with steroids, azathioprine and/or cyclosporin leads to improved LVEF, less hospitalizations, less need for heart transplantation, or death in patients with chronic inflammatory cardiomyopathy (23–26). Targeting CD20^+^ B-lymphocytes with the CD20 antibody rituximab improved hemodynamics in a series of patients with inflammatory DCM or cardiac sarcoidosis (27, 28).

Animal models of myocarditis and cardiomyopathies have provided insight into the role of inflammation in such entities. The primary models used were autoimmune and coxsackievirus B3 (CVB3) murine models (29, 30). They revealed that the primary immune infiltrate in the heart during acute myocarditis in viral and autoimmune models, as well as in patient biopsies, are macrophages, followed by smaller percentages of T and B cells and other cell populations like natural killer, dendritic and mast cells (19, 20, 31, 32). Neutrophils are important in the pathogenesis of myocarditis, but they appear in the heart early after infection or damage and so are not often observed in EMB. Innate immune factors that are critical in driving acute myocardial inflammation and progression to chronic inflammatory cardiomyopathy such as complement, Toll-like receptor (TLR)2 and TLR4, inflammasome components like NLR-family pyrin domain-containing protein 3 (NLRP3) and cytokines including IL-1β, IL-18, IL-6 and transforming growth factor (TGF)β1 are released from mast cells and macrophages (6, 19, 20). Mast cells are often overlooked in cardiomyopathy, but they are critical for remodeling to occur as they release most of the enzymes needed to activate the cytokines and other factors involved in remodeling and fibrosis (15, 33, 34). Mast cell degranulation is also typically associated with pericarditis, which occurs frequently during myocarditis in animal models. Remodeling and fibrosis along the pericardium are important drivers of progression from myocarditis to DCM (21, 35).

However, T cells also play a critical role in the pathogenesis of inflammatory cardiomyopathy and are frequently found in EMB (36). Importantly, the chronic phase of cardiomyopathy is an endpoint with significantly fewer immune cells, whilst more T cells are present (37).

In a mouse model of CVB3 myocarditis, CD8^+^ depletion had no significant effect on disease progression (38). Furthermore, CVB3-induced cardiac injury and prevention of chronic myocarditis was found to be unrelated to perforin-mediated cytotoxicity in mice (39). T cells and T cell derived cytokines including IL-4 and IL-17 seem to play a major role in promoting chronic inflammatory cardiomyopathy (38, 40–44). Although interferons (IFNs) such as IFNγ increase acute myocarditis, they might also be of protective value, as they decrease viral replication and protect against chronic inflammatory cardiomyopathy by inhibiting profibrotic factors including IL-4 and IL-17 (21, 41, 44–47). CD4^+^ CD25^+^ FoxP3^+^ regulatory T cells (T_reg_) might have protective function, as a low number of T_reg_ cells has been associated with worse DCM in autoimmune myocarditis (44, 48). Additionally, in viral and autoimmune models of myocarditis, females, as they have higher levels of T_reg_ cells and other regulatory factors such as T cell Ig mucin (Tim)-3 and IL-4, exhibit notably lower rates of myocarditis compared to males (32, 49). Administering T_reg_ cells prophylactically in a CVB3-induced myocarditis mouse model conferred protection against CVB3-induced myocarditis by exerting anti-inflammatory and antifibrotic effects (50).

There is mounting evidence that a detailed understanding of specific cell-cell interactions in myocarditis and inflammatory cardiomyopathy as well as involved signaling molecules will be paramount in the development of targeted novel therapies.

This narrative review aims to provide a comprehensive overview of the current knowledge on immune cells infiltrating the myocardium in myocarditis and non-ischemic cardiomyopathies that may be used as a resource for scientists investigating targeted cell-based therapies for cardiomyopathies.

## 2 Inflammatory cardiomyopathies

### 2.1 Viral myocarditis

#### 2.1.1 Cardiotropic viruses

##### 2.1.1.1 Enteroviruses

Myocardial infiltration is observed following infection by different coxsackieviruses via the coxsackievirus-adenovirus receptor (CAR) (51). CVB3 virus RNA is recognized by TLR3, TLR4 and melanoma differentiation-associated protein (MDA)-5 (52). Damage-associated molecular patterns (DAMPs) initiate the immune response from antigen presenting cells including mast cells, macrophages and dendritic cells. In that context, histamine, complement, and IL-1β activate infiltration of a diverse range of immune cells to the heart (6), including monocytes (6), natural killer cells (53), neutrophils (54), lymphocytes (55), and macrophages (55, 56). Macrophages are the dominant immune infiltrate in males with lower levels of CD4^+^ and CD8^+^ T cells as well as γδ T cells (56), B cells (57) and mast cells (20, 32). Females have higher levels of T and B cells as well as T_reg_ compared to males (58). B cells might play a role in progression to chronic disease, especially in females and autoimmune myocarditis (57), while T_reg_ cells might protect from progression to chronic myocarditis (53). Furthermore, Ly6C^high^ monocytes are detected in EMBs (59). Ly6C^high^ monocytes play a crucial role in early inflammation during acute myocarditis and are recognized for their ability to produce abundant levels of proinflammatory cytokines such as IL-1β (60–63). Additionally, they possess phagocytic capabilities (60–63).

Also in enterovirus myocarditis, the effect of IFNβ therapy has been explored (64). As stated earlier, IFNs including IFNβ and IFNγ reduce CVB3 replication and remodeling, which prevents progression to chronic cardiomyopathy (46, 47). Elevated IFN responses are a key reason that C57BL/6 mice do not progress from myocarditis to DCM in animal models of myocarditis (46, 47). Other studies explored pocapavir, pleconaril and IVIG in neonates with enterovirus myocarditis (65–67). Consensus statements, however, do not recommend antiviral therapies in enterovirus myocarditis as there is not sufficient evidence (3).

Soluble anti-CAR antibody reduces the incidence of acute and chronic CVB3-induced myocarditis in mice by preventing viral infection (68, 69). In another study, progression to chronic cardiomyopathy was prevented by anti-mouse IL-1β therapy (70). A primary pathway increasing acute inflammation and promoting progression to DCM in white background male mice is the TLR4-induced IL-1β response (20, 70–72). IL-1β increases IL-6 that is needed for IL-17/Th17 responses that promote remodeling and chronic cardiomyopathy (73). Male mice had increased IL-17-related responses compared to females and showed more fibrosis leading to chronic cardiomyopathy in CVB3-induced myocarditis (74). However, IL-17 does not increase acute myocarditis but is important for the progression to chronic cardiomyopathy which also predominantly occurs in males (20, 75, 76). Additionally, in acute murine CVB3 myocarditis high expression levels of osteopontin were found to be associated with the development of extensive fibrosis that can be reduced by treatment with a vitamin D analog (77). A recent study demonstrated that administering eplerenone can regulate the acute immune response and protect against myocardial remodeling in CVB3-induced myocarditis (78). Similarly, we had previously shown in an animal model of myocardial infarction that inflammatory genes activated during ischemia were downregulated through treatment with eplerenone (79). This response to treatment was much greater in females (79).

#### 2.1.2 Vasculotropic viruses

##### 2.1.2.1 Parvovirus B19

Parvovirus B19 (B19V) infects endothelial cells (80) which may lead to apoptosis of cardiomyocytes (80, 81). Only high copy numbers of B19V DNA in the myocardium were found to be associated with acute lymphocytic myocarditis (82). It is likely that the toxic, non-structural viral protein NS1 triggers the release of proinflammatory cytokines (83), although the pathogenetic role of B19V in myocarditis is still controversial (84). Immune cell infiltration in patients with Parvovirus B19V myocarditis is dominated by macrophages and lymphocytes (85). CD4^+^ T cells play a role in acute Parvovirus B19V-related myocarditis (86). In individuals, striking CD8^+^ T cell responses were observed, which were sustained or even increased over many months after the resolution of acute disease (87).

Although no guidelines currently recommend the use of IVIG for patients with severe Parvovirus B19V viremia and associated complications such as transient aplastic crisis or chronic pure red cell aplasia, some studies suggest that IVIG may be beneficial in such cases (88). Other authors showed no significant improvement in cardiac systolic function in patients undergoing IVIG, thus rendering the potential benefits of IVIG uncertain (89). No therapy is recommended when B19V copy number is low and cardiac inflammation is absent in EMB (3). When EMB is positive for inflammation although B19V copy number is low, immunosuppressive therapy may be considered (90).

New therapeutic strategies are under investigation, including the synthetic nucleotide analogues cidofovir and brincidofovir as well as flavonoid molecules and hydroxyurea (88).

#### 2.1.3 Lymphotropic viruses

##### 2.1.3.1 Human cytomegalovirus (HCMV)

HCMV is usually acquired during childhood and is known to infect several cell types, such as endothelial cells, epithelial cells, and immune cells (91, 92). Usually, HCMV infection remains asymptomatic until the occurrence of immunosuppression, thereby posing a significant concern as a complication in individuals such as organ transplant recipients or Human Immunodeficiency Virus (HIV) patients (92).

Male BALB/c mice infected with murine CMV (MCMV) develop acute and chronic inflammatory myocarditis similar to CVB3 and autoimmune models, where macrophages predominate with fewer T-, B-, and other cells during the acute phase of the disease (37, 93, 94).

A reduction of viral burden might be achieved with the anti-herpesvirus drugs ganciclovir or acyclovir, although the efficacy in HCMV-induced myocarditis has not been studied directly (95, 96). Viral infection was reduced in MCMV-induced myocarditis if ganciclovir and cidofovir were administered during the innate immune response but not if it was given during acute myocarditis (97). A controlled trial suggested that a CMV hyperimmunoglobulin treatment may be effective in HCMV myocarditis (98). Antiviral therapy is not generally recommended for patients with virus-induced myocarditis and should be reserved for individual cases (3). Consultation with an infectious disease specialist is recommended before initiating antiviral therapy (3).

##### 2.1.3.2 Epstein-Barr virus (EBV)

EBV invades cardiac tissue by initially infecting resting human B lymphocytes (96) and eventually infiltrating both the myocardium and pericardium (99). EMB from patients with EBV myocarditis typically reveals lymphocytic infiltration, consisting of predominantly CD8^+^ T cells but also CD4^+^ T cells (99). In this case, high numbers of EBV-encoded RNA copies were demonstrated in CD8+ T lymphocytes (99).

##### 2.1.3.3 Human herpesvirus 6 (HHV6)

HHV6-induced myocarditis has been associated with a myocardial infiltration of CD4^+^ and CD8^+^ T cells (100). Figure 2 illustrates EMB findings representative for HHV6-associated lymphocytic myocarditis. In a case report, Rohayem and colleagues reported a patient suffering from lethal acute B19V and HHV6 coinfection who at autopsy was found to have diffuse infiltration of the myocardium with mononuclear cells and neutrophils as well as edema, degeneration and loss of myocardial cells (101). However, low HHV6 copy numbers are a common finding and are likely to have no clinical impact (102).

#### 2.1.4 Cardiotoxic viruses

##### 2.1.4.1 Hepatitis C virus (HCV)

Chronic HCV infection may lead to cardiomyocyte hypertrophy and therefore hypertrophic cardiomyopathy due to expression of the HCV-core protein and overactivation of transcription factor AP-1 (103). The HCV-core protein plays a significant role in the viral nucleocapsids and has an impact on the transcription of cellular protooncogenes in hepatocytes (103, 104). HCV is also recognized to contribute to the development of insulin resistance and the generation of reactive oxygen species (105). The mechanisms that cause damage in relation to HCV-associated cardiomyopathy are not well understood. Hypotheses aiming to explain myocardial damage include direct mitochondrial disruption and oxidative stress to cardiomyocytes and a chronic systemic inflammatory state, which might lead to myocardial inflammation (103, 106). Furthermore, HCV core protein may lead to the activation of profibrotic pathways eventually leading to cardiac fibrosis (103, 106). In HCV-associated myocarditis, monocytes and CD68^+^ macrophages are the dominating cells infiltrating the heart (107).

HLA-DPB1*0901 and HLA-DRB1*1201 are associated with progression to chronic cardiomyopathy and persistence of HCV as well as the development of DCM (106). Mononuclear cells (in particular monocytes and CD68^+^ macrophages) are the primary target cells for treating extrahepatic manifestations in HCV infections (107).

Even though the viral genome typically persists in the myocardium after HCV infection, immunosuppression may be beneficial for HCV myocarditis, as it was shown to improve cardiac function (108).

##### 2.1.4.2 Human immunodeficiency virus (HIV)

In some cases of HIV-associated cardiomyopathy, diffuse myocardial damage with variable degrees of hypertrophy and degenerative changes leading to hydropic changes within the cardiomyocytes has been noted (109, 110). The HIV gp120 protein mediates myocardial injury and dysfunction through a nitric oxide dependent mechanism (111). Interstitial and endocardial fiber damage is increased, leading to fibrosis (109, 110, 112). T cells dominate the inflammatory infiltrate, with a majority of CD8^+^ T cells (109).

##### 2.1.4.3 Influenza viruses

Cytokine-mediated cardiotoxicity and autoimmune response against components of the heart are likely involved in myocarditis following influenza virus infection (113). Monocytes, dendritic cells, and macrophages dominate the myocardial immune infiltration. The latter two secrete cytokines such as IFNs and TNF-α, contributing to increased acute inflammation (114, 115).

#### 2.1.5 Angiotensin-converting enzyme 2 (ACE2)-tropic viruses

##### 2.1.5.1 Middle East respiratory syndrome coronavirus (MERS-CoV)

MERS-CoV-associated cardiomyopathy may be associated with myocyte hypertrophy, moderate coronary atherosclerosis and patchy myocardial fibrosis (116). There is limited data regarding myocardial immune infiltration. Lymphocytic infiltration (117, 118) predominantly with dendritic cells (119), macrophages (120), and T cells (121) has been described in MERS-CoV-induced myocarditis.

##### 2.1.5.2 Severe acute respiratory syndrome coronavirus (SARS-CoV)

Edema and atrophy of myocardial fibers is commonly seen in myocarditis associated with SARS-CoV infection (122). Immune infiltration primarily consists of lymphocytes. Also, monocytes and plasma cells infiltrating the endothelium have been described (122). As the ACE2 receptor is the entry point for SARS-CoV into cells, the protein might represent a therapeutic target (123).

##### 2.1.5.3 SARS-CoV-2

The role of SARS-CoV-2 on myocarditis was recently reviewed (124). Briefly, autopsy and histopathological findings suggest extensive lymphocytic infiltration in the myo- and pericardium after lethal SARS-CoV-2 infection (125, 126). An inflammatory state with predominantly CD68^+^ macrophages (124, 127) as well as enhanced monocyte recruitment (128) has been reported for cardiomyopathy associated with SARS-CoV-2 infection. Furthermore, a cytokine storm, dominated by IL-1β has been shown to play a major role in the pathophysiology of severe corona virus disease 2019 (COVID)-19 (129–131). IL-6 has been associated with cardiac dysfunction, as evidenced by reduced left ventricular function obtained with speckle tracking echocardiography in patients hospitalized due to COVID-19 (124, 132).

ACE2 receptor (124, 133) and transmembrane protease serine subtype 2 (TMPRSS2) (96, 124) interaction enables SARS-CoV-2 to enter its target cells (133). The interaction with the ACE2 receptor has been suggested as a potential direct cytotoxic effect of SARS-CoV-2 (133). While the possibility of direct damage to the heart by SARS-CoV-2 has been discussed (124), the prevailing absence of the virus within cardiomyocytes of COVID-19-associated myocarditis patients (134, 135) further reinforces the concept of cytokine-induced damage to the heart.

Several antiviral therapies are currently being investigated, such as protease inhibitors (e.g., lopinavir-ritonavir, darunavir), RNA polymerase inhibitors (remdesivir) and anti-cytokine agents (e.g., IL-6 receptor antagonists) (96). In patients with SARS-CoV-2, the IL-1β antagonist canakinumab improved clinical recovery and reduced cardiac injury at 28 days post infection (136, 137).

### 2.2 Bacterial myocarditis

#### 2.2.3 Borrelia-associated myocarditis

Borrelia species-induced myocarditis are associated with focal necrosis, hypertrophy and vacuolization of myocytes leading to fibrosis (138). Immune infiltrates are usually lymphocytic in nature (138), mostly composed of macrophages and lymphocytes (139), predominantly T cells (138). Mononuclear leucocytes were also seen in heart tissue (138).

##### 2.2.1.1 Staphylococcus

Staphylococcus typically builds micro abscesses within the myocardium (140). Methicillin-resistant *Staphylococcus aureus* (MRSA)-induced myocarditis is characterized by focal myocyte necrosis (141).

##### 2.2.1.2 Streptococcus

The pathomechanisms of myocarditis following streptococcal upper airway infections are not well understood. Streptococcal toxins and cross-reactivity of IgG with streptococcus antigens and cardiac myosin have been postulated as possible causative mechanisms (142, 143). Mononuclear cells, especially lymphocytes (of which CD4^+^ T cells were predominating) are found in EMB (144). Neutrophil infiltration and micro abscesses containing bacteria have also been described (145).

##### 2.2.1.3 Pneumococcus

Pneumococcus microlesions appear widely spread throughout the myocardium, but especially in the ventricles (146). Such microlesions manifest as areas within the myocardium with a reduced number of cardiomyocytes accompanied by an enlarged intercellular space filled with pneumococci (147). These lesions are further characterized by expansion of the intercellular space caused by extracellular vacuolation, the apparent loss of cardiomyocytes and the stark absence of infiltrating immune cells, as observed in both a BALB/c mouse model and human disease (147–149). Fibrosis is a common consequence of this response (148, 149).

Macrophages and neutrophils are subverted through biofilm production by pneumococcus (150).

Bacterial adhesin choline-binding protein A and its interaction with laminin receptor of vascular endothelial cells represents the cellular entry point for pneumococci (151). Also, cobinding of phosphorylcholine residues on the bacterial cell membrane to platelet-activating factor receptor is an additional activating mechanism (151). Neutralizing these interactions could be a possible pharmacological approach (151).

##### 2.2.1.4 Meningococcus

Meningococcus myocarditis is rare (152), but might occur during meningococcus sepsis (153, 154). There is no information regarding immune cell infiltration patterns in the heart available, but neutrophils and lymphocytes accumulate around meningeal vessels of patients suffering from meningococcal meningitis (155).

##### 2.2.1.5 Gonococcus

Myocarditis caused by gonococci usually occurs secondary to or during endocarditis (156, 157) leading to perivalvular abscesses (156, 158). Neutrophils are the dominant immune cells infiltrating the valves and spatially related myocardium during such an infection (158). Fibrosis is a common result of gonococcal myocarditis (158).

##### 2.2.1.6 Salmonella

Following salmonella gastroenteritis, the heart and aorta may be a secondary target of inflammation (159). It remains uncertain whether this represents a sterile inflammation lacking bacterial myocardial infiltration, that occurs subsequent to the infection, or if it is a secondary manifestation directly linked to the salmonella infection (159). Non-typhoid salmonella might lead to multifocal biventricular inflammation in subepicardial and midmyocardial tissue (160). There is no data in the literature regarding the composition of the immune cell infiltrate.

##### 2.2.1.7 Mycobacterium tuberculosis

Infiltration by *Mycobacterium tuberculosis* of the heart follows no specific pattern (161). Similar to tuberculosis of the lungs, myocardial involvement comes in 3 different types: tuberculomas, miliary tubercles or an uncommon diffuse infiltrative type (161). Commonly, the left ventricle is affected (162). Usually, a giant cell (163–165) and lymphocytic (164, 165) infiltrate accompanies the infection. Granulation (166) as well as scar tissue and fibrosis might result (163).

*M. tuberculosis* inactivated antigens are used as the adjuvant (mimic infection) in complete Freund's adjuvant that is required to induce disease in autoimmune models of myocarditis (29). The dominant immune response in natural infections and in experimental autoimmune myocarditis are macrophages and Th17-type responses (167–169). These findings suggest that *M. tuberculosis* can drive myocarditis and DCM in the context of an autoimmune response.

##### 2.2.1.8 Mycoplasma pneumoniae

Myocarditis due to *Mycoplasma pneumoniae* infection has been described as myopericarditis (170). Fibrosis of the heart valves has been reported (171). Data are limited concerning EMB findings on cellular components of the immune infiltrate in the context of mycoplasma infection.

##### 2.2.1.9 Brucella

Brucella myocarditis is very rare (172) and tends to occur in the left ventricular myocardium, in particular subpericardial (173, 174). Following infection with Brucella, endocardial tissue might undergo calcification and become fibrotic (175). Data are limited regarding the type of immune infiltration.

#### 2.2.2 Others

Several other infections such as protozoa, fungi or parasites can cause myocarditis (3). As there is limited data currently available on specific inflammatory infiltrates in most of these entities, only the most common parasite causing myocarditis, *Trypanosoma cruzi,* is discussed.

##### 2.2.2.1 *Trypanosoma cruzi*—chagas disease

Chagas disease is caused by *Trypanosoma cruzi*, which is a parasite that replicates within host cells, including cardiac myocytes (176). Cardiac involvement in Chagas disease typically leads to inflammation, fibrosis, diffuse ventricular wall motion abnormalities and arrhythmias especially when prolonged inflammation occurs (177, 178). The myocardium shows signs of necrosis, areas of myocellular hypertrophy and predominantly mononuclear cell infiltration (176). Macrophages, eosinophils, neutrophils and mast cells are found in myocardial tissue as well, but to a lesser extent (176). Proinflammatory cytokines such as TNFα and IFNγ promote inflammation which can further lead to autoimmunity (179). Furthermore, the persistence of the parasite is associated with high grade myocarditis (180), as tissue damage partly results directly from the pathogen itself (176).

### 2.3 Immune-mediated myocarditis

#### 2.3.1 Rheumatoid arthritis

Rheumatoid arthritis associated cardiomyopathy is characterized by fibrosis and citrullination within the myocardial intercellular space (181). The latter is a type of post-translational modification that converts arginine residues within proteins to citrulline residues (182). In rheumatoid arthritis, the immune system produces antibodies targeting those citrullinated proteins (182–185). Rheumatoid arthritis associated myocarditis is characterized by focal necrosis or granulomatous inflammation (186). There is no specific immune cell type dominating the immune infiltrate.

#### 2.3.2 Vasculitis

1.5% of patients with myocarditis are found to have necrotizing coronary vasculitis by EMB (187). In the case of eosinophilic granulomatosis with polyangiitis eosinophilis play the dominant role (188). As of today, data on the components of cellular infiltrates in other entities is very limited.

#### 2.3.3 Other connective tissue diseases

Myocarditis associated with connective tissue diseases such as systemic lupus erythematosus develops predominantly in the ventricular wall leading to edema and necrosis (189, 190). CD4^+^ T cells usually dominate the immune infiltrate (189).

Severe combined immunodeficient mice developed myocarditis dominated by CD4^+^ T cells, while depletion of CD4^+^ T cells suppresses the inflammation (189). In mice with myocarditis induced through the transfer of CD4^+^ T cells, Th1 and Th17 cells were found to infiltrate the myocardium (189).

#### 2.3.4 Cardiac sarcoidosis

Cardiac sarcoidosis primarily leads to subepicardial inflammation within the left ventricular septum. However, midmyocardial, subendocardial or transmural inflammation may also occur (191). Infiltration is predominantly granulomatous (192).

Th1 cell derived cytokines such as IL-2, IFNγ and IL-12 drive the granulomatous inflammation, while the infiltrate lacks Th2 cells (192–194). In a case report, Schoppet et al. described a high Th1 response as causative for multiple granulomas in EMB while a Th2 response has been associated with disease regression (195). Figure 3 illustrates EMB findings representative for cardiac sarcoidosis.

Cytokines derived from Th1 cells promote macrophage accumulation, with CD68^+^ CD163- M1 macrophages dominating (196–198). Macrophages play a major role in the formation of granulomas (192). In the early stages of the disease, macrophages tend to form multinucleated giant cells within granulomas of the foreign body type (192). In later stages, they form granulomas of the Langhans type (192). In the EMB, the presence of Schaumann bodies and asteroid bodies may be observed (192). Schaumann bodies are calcified protein structures that occur intracellularly, often within giant cells (192, 199). In contrast, asteroid bodies are comprised of non-collagenous filaments and myelinoid membranes and are also frequently found in multinucleated giant cells (192, 199). Both Schaumann and asteroid bodies are pathologic signs that may be found in sarcoidosis (192, 200).

M2 macrophage derived factors, such as TGF-β and chemokine CC motif ligand lead to fibrotic cardiac remodeling (196, 201, 202).

Anti-inflammatory treatment with glucocorticoids is recommended and other anti-inflammatory agents such as TNF*α* antibody can be considered referring to current consensus statements (203).

#### 2.3.5 mRNA vaccine related myocarditis (against SARS-CoV-2)

This topic has been recently reviewed (31, 124). Briefly, SARS-CoV-2 vaccine-related myocarditis is associated with myocardial edema and mostly subepicardial anterolateral and inferolateral involvement of the myocardium (204). A number of theories have been proposed for how vaccines, and mRNA vaccines in particular, could lead to myocarditis including innate immune activation of mast cells (124), myocyte necrosis (205, 206), cytokine-induced damage (207), and interstitial fibrosis (208). In EMB, SARS-CoV-2 vaccine-related myocarditis usually presents as lymphocytic myocarditis (209), although in some biopsies no inflammatory cells are found (204, 209). The inflammatory infiltrate primarily consists of macrophages and T cells (205, 206).

Various mechanisms underlying myocarditis following SARS-CoV-2 vaccination including cytokine related damage and spike glycoprotein antibodies cross-reacting with myocardial contractile proteins, as well as the influence of sex and gender through endocrine differences are discussed (207, 210). In young men, autoantibodies against the IL-1RA impairing the IL-1RA bioactivity *in vitro* were associated with low circulating levels of IL-1RA and were found in patients with biomarker evidence of cardiac damage and inflammation (211).

#### 2.3.6 Autoimmune myocarditis

Autoimmune myocarditis may follow ischemic (212), surgical (213) or traumatic (214, 215) myocardial damage as a consequence of an immune response to released segregated antigens (i.e., cardiac myosin). Furthermore, autoimmune myocarditis has also been hypothesized to follow viral infections that release damaged heart tissue (37). The experimental autoimmune myocarditis (EAM) model closely follows the time-course, cellular infiltrate, sex differences and mechanisms of disease that have been identified with clinical myocarditis associated with viral infections (20, 216). Importantly, autoimmune models of myocarditis that use a mild viral infection (i.e., MCMV, CVB3) instead of complete Freund's adjuvant as the pathogen strongly mimic EAM and clinical viral myocarditis (20, 37, 124). Recognition of autoimmune myocarditis can be crucial for initiation of immunosuppressive therapy (217) with potential recovery of cardiac function.

### 2.4 Toxicity-Induced myocarditis

#### 2.4.1 Alcoholic cardiomyopathy

The direct toxic effect of ethyl and acetaldehyde leads to ultrastructural alterations of the mitochondria and sarcoplasmatic reticulum of cardiac myocytes (218, 219). Protein metabolism is dysregulated at a molecular level leading to arrhythmias (218) and focal necrosis (218, 220). This causes a lymphocytic infiltrate, without a subtype of cells dominating (218, 221).

Chronic alcohol consumption directly influences immune cell concentration with a specific suppression of neutrophils (222–224). Alcohol use disorder (AUD) might also reduce lymphocytes (224–228) and their reactivity to mitogens (224, 229). Activation of T and B cells might be aggravated due to heightened antigen presentation (224, 230). After alcohol exposure T cells produce less IFN-γ (224, 231).

#### 2.4.2 Drug-induced cardiomyopathies

Oxidative stress that disrupts mitochondria and reduces ATP production is the main cause of drug-induced injury to the myocardium (219). In addition, reactive oxygen species interfere with mitochondrial DNA replication, which can further lead to myocardial damage (219, 232). In a postmortem examination, hearts of patients who developed cardiotoxicity as a result of cyclophosphamide medication featured hemorrhagic myocardial necrosis, interstitial edema, hemorrhage and fibrin deposition (233, 234). However, the precise mechanism of cyclophosphamide-induced injury to the myocardium is not yet clear (233, 234), but inflammatory cell infiltrates have been described (232). Treatment with doxorubicin also damages mitochondria and leads to a reduction of natural killer cell activity and stimulation of cytotoxic T cells (235). A decrease in macrophage differentiation has also been observed (235). EMB shows histiocytes dominating the infiltrate in anthracycline-induced cardiomyopathy (236, 237). Several other drugs are known to cause myocarditis rarely, such as antibiotics, diuretics and antidepressants (3).

#### 2.4.3 Immune checkpoint inhibitor (ICI)-induced cardiomyopathy

The prevalence of ICI-induced myocarditis revolves around 1% in a multicenter registry and is generally considered underestimated (238). ICI-induced myocarditis is associated with a mononuclear, mainly lymphocytic infiltrate within the myocardium, myocyte degeneration (239, 240) and interstitial fibrosis (241). T cells are the dominating cellular phenotype (239, 240, 242), with similar levels of CD4^+^ and CD8^+^ T cells (239, 240). CD68^+^ macrophages are also involved, and antibody deposits might occur (239, 240).

In an A/J mouse model, Won et al. were able to show that treating naive mice with anti-programmed cell death protein (PD)-1 monoclonal antibody (PD-1 regulates/inhibits T cell responses) was able to induce myocarditis (243). Tumor cells or infectious agents were absent in this model (243). Troponin elevation, arrhythmias and lymphocytic infiltration of the myocardium was observed in this model (243). The investigators identified that T cells being reactive to cardiac myosin were elevated in the myocardium (243).

Lv et al. demonstrated that autoimmunity plays a role in ICI-induced myocarditis by demonstrating that CD4^+^ T cells specific for alpha myosin heavy chain were not effectively eliminated in the thymus of mice and humans after receiving immune checkpoint inhibitors (244). They identified high numbers of these autoreactive T cells in mice and in patients with myocarditis (244).

ICI therapy disrupts cytotoxic T-lymphocyte-associated protein (CTLA)-4 and PD-1 signaling on T cells, leading to a reduction in peripheral immune tolerance and an increased likelihood of T cell activation (242, 245, 246). Another possible mechanism for ICI-associated myocarditis is the proliferation of T cells that recognize an antigen shared by both the tumor and heart muscle (242, 245, 246).

In a further study, researchers observed that individuals with ICI myocarditis had an increased number of cytotoxic CD8^+^ T cells that expressed the CD45RA marker, which is found on naive CD8^+^ T cells (247). This contrasted with healthy individuals, who had lower levels of these cells (247). Sequencing of the T cell receptor indicated that the CD8^+^ CD45RA^+^ T cells were clonally augmented in patients with ICI myocarditis (247). An analysis of the gene expression patterns in Temra CD8^+^ T cells revealed that they have a cytotoxic and activated phenotype, as expected (247). Their interaction with innate immune cells was enhanced and anti-inflammatory regulating factors were missing, leading to more inflammation (247).

## 3 Non-ischemic non-inflammatory cardiomyopathies with background inflammation

### 3.1 Hypertrophic cardiomyopathy (HCM)

In HCM an accumulation of CD8^+^ T cells (248, 249), basophils, fibroblasts, and platelets has been described in the cardiac inflammatory infiltrate (248). Analysis of the immune infiltration in HCM patients vs. controls revealed a decrease in dendritic cells, macrophages, monocytes, and natural killer cells (249). A decrease in CD163 ^+ ^LYVE1^+^ macrophages, which belong to the M2 macrophage subtype, may play a crucial role in the pathogenesis of the disease (248). Other authors have demonstrated a high abundance of neutrophils, as well as both naive and memory B cells, within the myocardium (250).

### 3.2 Peripartum cardiomyopathy

EMB of myocardial tissue in patients with peripartum cardiomyopathy revealed evidence of a mild cardiac infiltration of inflammatory cells; however, no discernible pattern of cells was described (14). Breastfeeding women were found to have increased levels of prolactin, which were correlated with heightened levels of CD8^+^ T cells in the circulation (251). The inhibition of prolactin release using the dopamine antagonist, bromocriptine, prevents the development of peripartum cardiomyopathy in mice, suggesting a potential role of prolactin in the pathogenesis of the disease (252, 253). McTiernan et al. reported a significant reduction in circulating natural killer cells, along with elevated levels of CD3^+ ^CD4^−^CD8^−^CD38^+^ T cells in women with peripartum cardiomyopathy (254).

Recently, bromocriptine, a prolactin release inhibitor has been explored as a therapeutic option in peripartum cardiomyopathy. It has been shown that bromocriptine treatment improves LVEF in peripartum cardiomyopathy patients (255–259). The decrease of prolactin might reduce the prolactin-associated inflammation and might therefore explain the observed increase in LVEF.

### 3.3 Arrhythmogenic cardiomyopathy

The presence of patchy inflammatory infiltrates suggests a potential involvement of the immune system in the pathogenesis of arrhythmogenic cardiomyopathy (260, 261), with M1 and M2 macrophages in equal numbers dominating the lesion (262). Cardiac myocytes in Dsg2mut/mut hearts demonstrated positive immunoreactivity for IL-1β, TNFα, and MCP-1α (263). Additionally, infiltrating mononuclear inflammatory cells showed positive immunoreactivity for IL-1β and TNF*α* (263). Cardiac inflammation leads to fibrotic remodeling of the myocardium in a murine model of arrhythmogenic cardiomyopathy with a desmoglein 2 mutation (262). Clinically, affected individuals display replacement of the ventricular myocardium with fibrofatty tissue (260, 261, 264–266). The implementation of therapy aimed at inhibiting remodeling and fibrosis could potentially improve cardiovascular function and might limit disease progression but requires further investigation (267).

### 3.4 Takotsubo cardiomyopathy

Fibrosis is a characteristic feature observed in EMB samples obtained from the myocardium of patients with Takotsubo Syndrome (TTS) (268). Clinical studies have documented the infiltration of monocytes and macrophages in myocardial tissue (268–270). According to single-cell RNA sequencing studies analyzing immune cells in myocardial tissue, TTS-like cardiomyopathy is associated with intricate activation of both innate and adaptive immune cells in the heart (268). Among these cells, macrophages were found to be predominate (268). When global macrophage depletion was induced through clodronate liposome administration or macrophage infiltration was blocked using a CCR2 antagonist or in CCR2-KO mice, cardiac function was improved in mice challenged with isoproterenol (268).

### 3.5 Metabolic cardiomyopathy

#### 3.5.1 Diabetes mellitus

In diabetes, various pathophysiological factors contribute to the development of cardiomyopathy, such as systemic metabolic disturbances, inappropriate activation of the renin-angiotensin-aldosterone system, subcellular component abnormalities, oxidative stress, inflammation, and impaired immune modulation (271–273). During obesity or insulin resistance, there is a notable occurrence of macrophage polarization, particularly towards M1 macrophages, as well as activation of dendritic cells and T lymphocytes (271, 273). Recent studies have provided evidence suggesting that B cells have a significant impact on type 1 diabetes and its associated complications (274). High rates of B-cell depletion have been shown to delay the progression of disease-related pathology in non-obese type 1 diabetic mice and new-onset patients with the disease (274).

Administration of glucagon-like peptide-1 for a duration of 5 weeks resulted in improvement in LVEF and functional status in patients with chronic heart failure and comorbid diabetes, which suggests that insulin resistance is implicated in the pathogenesis of cardiomyopathy in diabetes mellitus (275). Those findings require further investigation and are therefore not implemented in current clinical practice.

#### 3.5.2 Gouty myocarditis

Among metabolic entities, myocarditis causing cardiac dilation and dysfunction with heart failure is a possible manifestation of gout, particularly in patients with severe and untreated forms (276). Amorphous urate crystals can deposit inside the cardiomyocytes and induce a strong inflammatory response associated with macrophages and cell death (276). Activation of TLR4, NLRP3 and IL-1β in gout are known factors that drive the pathogenesis of myocarditis (277).

### 3.6 Fabry disease

Fabry disease is characterized by glycosphingolipid (Gb) accumulation in cells, leading to a proinflammatory response that may impact disease progression and causes myocardial edema (278). In some cases, small coronary vessels, conduction tissue and subepicardial ganglia were infiltrated (278, 279). CD3^+^ T lymphocytes were identified as the primary component of the observed inflammatory infiltrates (278).

The chronic secretion of highly immunogenic Gb3 by affected Fabry cells may play a key role in the initiation of immune-mediated myocardial inflammation and subsequently cause interstitial damage (279).

### 3.7 Cardiomyopathies associated with neuromuscular diseases

The dystrophinopathies are a heterogenous group of X-linked neuromuscular disorders associated with cardiomyopathy and altered cardiac immune regulation (280). In Duchenne muscular dystrophy-associated DCM, pentraxin 3 has been shown to act as an inflammatory mediator, facilitating the functions of macrophages and dendritic cells and contributing to apoptosis and necrosis (281).

## 4 Discussion

In this review, we provide a comprehensive summary of the current literature on the immune landscape of myocardial diseases specific to their causes. However, for many types of myocarditis and inflammatory cardiomyopathy little data is available based on histology from EMBs and there are no corresponding translational animal models to better understand the pathogenesis of disease.

We highlight in this review that many infections, in particular viral, and many different chemicals/drugs are able to induce myocarditis and inflammatory cardiomyopathy. Most of our understanding of the mechanisms that drive the pathogenesis of myocarditis and inflammatory cardiomyopathy or DCM was derived from animal models.

An EMB provides a snapshot of immune cells located within the myocardial tissue at the time of sample collection. Furthermore, EMB does help with the definite identification of etiologies such as giant cell myocarditis in which EMB findings help to initiate anti-inflammatory treatment (282, 283). In giant cell myocarditis, EMB is further utilized for prognosis estimation (282, 283). Although being helpful in the identification of patients who might benefit from anti-inflammatory treatment, an EMB does not provide information about pathophysiological processes within other areas of the heart and may not be obtained during the peak of myocardial inflammation in the contrary to animal models, where timing of sample collection can be very accurately determined. Cardiac magnetic resonance imaging (CMR) is the non-invasive gold-standard to diagnose myocarditis. It primarily identifies edema and fibrosis rather than directly detecting activity of inflammation. Animal models have revealed that edema and fibrosis occur after the peak of acute myocarditis as the disease progresses toward cardiomyopathy and so EMBs that are obtained at the time when CMR findings become visible are potentially capturing a later timepoint in the pathogenesis of disease. We need to keep this in mind as we consider pathogenic mechanisms. Additionally, investigators testing for immune cells in EMBs may not stain for all key immune cell types that animal models have identified to be important in the pathogenesis of disease such as mast cells, macrophages and neutrophils which may lead to T cells being reported more often in EMBs.

In viral myocarditis, mast cell, macrophage and T cell responses and cytokines released thereof dominate as drivers of disease. The idea of innate cytokine storm-related damage to the myocardium has been discussed as one pathogenetic mechanism in severe COVID-19 but has long been known to be a factor in other forms of myocarditis. The innate cell release of TNF*α*, IL-1β and IL-6 as well as complement activation are well documented in viral and autoimmune models of myocarditis, especially in males, where they increase acute and chronic myocarditis (124, 129–131, 284, 285). IL-6 increased by TLR4-released IL-1β drives T cells to a Th17 response that contributes along with IL-1β to remodeling and fibrosis that leads to chronic cardiomyopathy/DCM. In general, these mechanisms are also imaginable in bacterial or toxic myocarditis, where neutrophils augment the adaptive T cell response. Furthermore, myocarditis associated with autoimmune diseases such as connective tissue diseases, have a greater T and B cell/autoantibody response that is increased by estrogen in females who primarily develop these conditions. ICI-induced myocarditis is also dominated by an autoimmune T cell response that is created by drugs, that disinhibit PD-1 signaling. More research is needed to understand the immune infiltrate in cardiovascular diseases that are not traditionally considered as “inflammatory cardiomyopathies”, such as HCM or arrhythmogenic cardiomyopathy.

With the growing relevance of precision medicine and cellular therapies, it is imperative to gain a comprehensive understanding of the precise immune mechanisms involved in myocardial inflammation, in particular of the cytokines involved and the patterns they form, as this knowledge can inform the development of novel therapeutic approaches. Major gaps include the need to obtain better clinical data on the early and chronic immune response to various viruses, toxins and other causes of disease. Additionally, myocarditis and chronic cardiomyopathy occur predominantly in males and a better understanding of the role of sex and gender differences in disease is needed. As sex influences the levels of T and B cells as well as macrophages (20, 32, 56–58), sex is needed to be taken into consideration when developing targeted therapies. With the advent of new technologies that can identify gene profiles in distinct cell populations from EMB and animal models, a better understanding of similarities and differences in cardiomyopathy phenotypes should emerge that will inform novel therapy development. Thus, this review not only provides an overview of the immune landscape in myocardial diseases but also identifies research gaps in our understanding of cardiac inflammation. By highlighting these gaps, we aim to stimulate further translational investigation into the pathogenesis of myocardial inflammation and ultimately facilitate the development of more effective treatments.

A critical question in the field of cardiac therapeutics concerns the identification of patients who may benefit from anti-inflammatory therapy. To address this, new diagnostic methods are required to enable early detection or screening for inflammatory components in patients with heart failure, thereby allowing for timely initiation of anti-inflammatory treatment. Magnetocardiography (MCG) might emerge as a promising diagnostic tool for this purpose, offering the capability to measure the cardiac magnetic field (286–288). The interplay of electrons and ions and the directions of their respective movements shape the cardiac magnetic field and may deviate in disease (286–288). Even discrete alterations within the electromagnetic field are quantifiable, since small quantum interference device sensors (SQUIDs) were introduced into magnetocardiography (287, 288). It is noteworthy that obtaining MCG measurements require only one minute per patient (287, 288). Being a non-invasive and passive diagnostic tool, magnetocardiography entails no side effects (287, 288) which further renders it a potent tool with diverse possibilities for future research and clinical applications. Currently, the utility of MCG is being explored in screening for inflammatory cardiomyopathy and assessing early treatment response to anti-inflammatory treatment in respective patients (286–290).
